# Pharmacological Hypotension as a Cause of Delirious Mania in a Patient with Bipolar Disorder

**DOI:** 10.1155/2017/2809205

**Published:** 2017-12-19

**Authors:** Manuel Glauco Carbone, Mario Miniati, Marly Simoncini, Beniamino Tripodi, Claudia Tagliarini, Claudia Carmassi, Liliana Dell'Osso

**Affiliations:** Department of Clinical and Experimental Medicine, University of Pisa, Via Roma 57, 56100 Pisa, Italy

## Abstract

Delirious mania is a severe but often underrecognized syndrome characterized by rapid onset of delirium, mania, and psychosis, not associated with a prior toxicity, physical illness, or mental disorder. We discuss the case of a delirious mania potentially triggered and maintained by a systemic hypotension induced by antihypertensive drugs. Symptoms recovered completely after the discontinuation of antihypertensive medications and the normalization of blood pressure levels.

## 1. Introduction

Delirious mania is a severe but often underrecognized syndrome characterized by rapid onset of delirium, mania, and psychosis, with no identifiable organic cause [1]. Literature describing its clinical characteristics and treatment is relatively sparse [2]. However, delirious mania needs attention because of its high morbidity and fatal outcomes and has to be managed vigorously. We describe a case report of a patient with a history of Bipolar I Disorder who suddenly developed a delirious mania that might have been triggered and maintained by the use of antihypertensive medications.

## 2. Case Presentation

Mr. A. is a 78-year-old man with a late-onset Bipolar I Disorder (DSM-5) that started at the age of 64. The onset was with a hypomanic episode characterized by easy irritability, anger, and hyperactivity in work and social life. No specific treatment was necessary at that time. The first manic episode was several years later, at the age of 76, with mood swings, psychomotor hyperactivity, increased levels of energy, and delusions. Mr. A. was hospitalized for the first time and treated with valproate (1000 mg/day). During the following months, Mr. A. showed mood instability with subclinical episodes of depressed mood, apathy, anhedonia, and clinophilia. However, he was no longer on pharmacological treatment. He was admitted again to a psychiatric hospital at the age of 78, after 3 months of apparent well-being. He had a manic episode, characterized by elevated and irritable mood, reduced need for sleep, pressured speech, racing thoughts, distractibility, disorganized behaviours, and auditory and visual hallucinations.

These symptoms started abruptly one week before hospitalization. His family reported that at home he showed fluctuating levels of consciousness and incoherent speech. He also had outbursts of anger and destructive behaviours. After hospitalization, Mr. A.'s symptoms persisted despite acute treatment with valproate (1000 mg/day), quetiapine (75 mg/day), and lorazepam (2.5 mg/day). As part of the clinical evaluation, the patient had a work-up to rule out medical or neurological causes for his condition. A specialist in internal medicine and a neurologist evaluated Mr. A. but no acute medical or neurological signs and symptoms were found. They decided to continue the medications he was taking at home for the diagnoses of hypertension, hypercholesterolemia, Barrett's oesophagus, and a mild benign prostatic hypertrophy. His medications included bisoprolol (2,5 mg/day), alfuzosin (10 mg/day), enalapril (20 mg/day), hydrochlorothiazide (12,5 mg/day), ticlopidine (250 mg/day), and pantoprazole (20 mg/day). A computed tomography (CT) scan was performed. Chronic ischemic changes and mild ventricular dilatation were found but considered as compatible with old age. Electroencephalographic (EEG) results showed poor alpha waves and mild cortical dysfunction. The neurologist concluded that these were findings frequently seen in elderly patients and not related to the acute condition. Acute metabolic syndromes were also excluded: all blood examinations, including blood count, serum glucose, sodium, potassium calcium, magnesium, chloride, and phosphates, were within normal ranges. Vitamin B12 and folate were also normal. Blood culture, urine culture, and VDRL (venereal diseases research laboratory) testing were negative. No substance use, abuse, or withdrawal were diagnosed. Neuroleptic malignant syndrome and serotonin syndrome were considered but excluded as no pyrexia, autonomic instability, or marked rigidity was detected. A diagnosis of delirious mania was made. Over the first seven days of hospitalization, Mr. A. showed a state of partial orientation, with fluctuating cognition, hyperactivity with purposeless activities. On better days, Mr. A. was able to answer the simpler questions with regard to orientation to place and person. In these intervals, the patient had nearly normal speech and thought processes with only occasional racing thoughts. During the worst days, he was noted mumbling to himself, apparently busy without clear goals, often shouting, and frightened by auditory and visual hallucinations. After 12 days of hospitalization, Mr. A. had a syncope episode with release of faeces and urine, followed by a quick recovery of consciousness. The neurologist was alerted again. An acute ischemic transitory attack was excluded. An EKG was performed together with the search for markers of heart necrosis but all tests were negative. The monitoring of pressure levels for the subsequent hours revealed that the patient had in clinostats average arterial blood pressure (PA) of 100/70 mmHg with an average heart rate of 78; in orthostats PA was 95/70 with average heart rate of 90. Most of the antihypertensive therapy was stopped, maintaining only bisoprolol (2.5 mg/day). A cardiac Holter, a pressure Holter, an echocardiogram, and an echo-colour-Doppler of the carotids were performed. The carotid echo-colour-Doppler showed a mild degree of bilateral stenosis, but again not significant from a clinical point of view.

In the subsequent 3 days, temporally related to a progressive rise in pressure values (stabilized around 130/80 with regular heart rate), and with no changes in psychopharmacological treatments, the patient showed a marked improvement, with reduction of psychomotor agitation and normalization of behaviour. Mr. A.'s irregular sleeping patterns were normalized and he was able to sleep through the night. The disorientation resolved completely. He became stable enough for discharge to outpatient care on quetiapine, valproate, and lorazepam at fixed dosages. After considering delirious mania and his lack of response to standard therapies, including lorazepam, we hypothesized that the resolution was due to the discontinuance in antihypertensive medications and to the subsequent normalization of blood cerebral perfusion.

## 3. Discussion

Mr. A. showed the typical features of delirious mania: rapid onset of disorientation, fluctuating sensorial patterns, altered level of consciousness, intense excitement, emotional lability, disorganized rambling speech, hallucinations, delusions, subtotal insomnia, and bizarre and disorganized behaviours, in the absence of identifiable organic causes. However, the clinical picture apparently was not influenced by standard psychopharmacological treatments and resolved only when the antihypertensive drugs were discontinued almost completely. We hypothesized two different mechanisms to explain this clinical course. First, the patient was in a chronic condition of overmedication with antihypertensive drugs not adequately targeted to the fluctuations of his blood pressure and cerebral perfusion, as demonstrated by the syncope. We hypothesized that a subjective abnormal sensitivity in blood pressure regulation in a patient with a history of Bipolar I Disorder might have led to recurrent episodes of transient cerebral hypoxia, ultimately triggering and maintaining a delirious mania. Data from literature shows that elderly patients, even if with no acute metabolic or hemodynamic dysfunctions, might have subthreshold and transient fluctuations of cerebral perfusion, leading to dopaminergic hyperactivity and cholinergic deficiency that might predispose to a delirious mania [3, 4].

The second hypothesis regards the potential neuropsychiatric effects of several antihypertensive drugs. Hallucinations, mania, euphoria, sedation, and transient disorientation have been described in patients receiving centrally acting antihypertensive drugs, such as beta-adrenergic blockers, in relation to their antiadrenergic and angiotensin-converting enzyme (ACE) inhibitor mechanisms [5]. Several reports have described complex neurotransmitter-related effects on brain beta-adrenoreceptors and serotonin (5-HT) receptors of antihypertensive drugs, especially in elderly patients [6, 7]. Occasionally, more serious reactions, including depression, vivid nightmares, dreams, hallucinations, delirium, and paranoid psychosis resembling acute schizophrenia have been reported following treatment with beta-blockers, mainly with propranolol [4, 8, 9]. Moreover, it has been shown that psychiatric effects of beta-blockers are not dose-dependent and are related to individual sensitivity [10]. Also, the more recent angiotensin-converting enzyme (ACE) inhibitor class of antihypertensive drugs has demonstrated many neuropsychiatric effects, such as triggering manic episodes, or unmasking subsiding psychotic symptoms [11, 12]. Recently, a specific role of the renin-angiotensin system has been confirmed as a possible new target for mood dysregulations, considering that available preclinical and clinical data seem to suggest the potential antidepressant properties of ACE inhibitors, even if definitive proof from RCTs is yet to come [13, 14].

It has been also hypothesized that the ACE protease inhibitor might block the enzyme enkephalinase that is important in the breakdown of enkephalins, with potential effects on behaviours [15, 16].

Taken together, all these reports raise questions on the need for the adoption of closer monitoring when antihypertensive drugs are administered to patients with a history of Bipolar Disorder, considering their sensitivity to the psychotropic effects of a number of medications. Unfortunately, delirious mania lacks formal diagnostic criteria, and most descriptions of this syndrome are from case series, with different courses and nonunivocal etiology. Even if Mr. A. recovered after the discontinuation of antihypertensive medications and the normalization of blood pressure levels, we cannot define a certain cause-effect relationship, despite the course evoking such an explanation. However, we believe that psychiatrists should be always aware that beta-blockers or ACE inhibitors can trigger a delirious mania episode in the elderly, especially in the presence of a history of Bipolar Disorder.
